# Rectal surgery for hirschsprung's disease in a single pediatric tertiary care center: improvement of rectal dissection following the introduction of robotic technology

**DOI:** 10.3389/fsurg.2026.1762703

**Published:** 2026-03-11

**Authors:** Maria Grazia Faticato, Serena Reali, Michela Cing Yu Wong, Stefano Avanzini, Girolamo Mattioli

**Affiliations:** 1 Pediatric Surgery Unit, IRCCS Istituto Giannina Gaslini, Genoa, Italy; 2 DINOGMI, University of Genova, Genoa, Italy

**Keywords:** hirschsprung's disease, minimally invasive surgery, proctocolectomy, pull-through, rectal dissection, robot-assisted, robotic

## Abstract

**Introduction:**

Several surgical techniques have been described in the literature for the treatment of Hirschsprung's disease (HD). This study presents our surgical experience in managing patients with HD and the evolution of rectal dissection following the introduction of robotic technology. We developed a novel laparoscopic robot-assisted approach and report our initial experience with Restorative Proctocolectomy with minimal transanal endorectal dissection in HD patients, detailing the technical aspects of this procedure.

**Patients and methods:**

We retrospectively reviewed data from patients with HD who underwent pull-through surgery at our Institution between January 2015 and July 2025.

**Results:**

Sixty-five patients with HD were included. Fifty-four patients underwent conventional procedures performed at our Center: two open Soave endorectal pull-through (ERPT), forty-four laparoscopic Soave-Georgeson ERPT, and eight Totally Robotic Soave ERPT with limited transanal endorectal dissection. Beginning in 2023, eleven patients underwent a robot-assisted Restorative Proctocolectomy with minimal transanal endorectal dissection.

**Conclusion:**

The advantages of robotic technology allow for safer performance of pelvic and particularly rectal surgery. Based on our experience, we suggest that in selected patients with HD, Restorative Proctocolectomy with minimal transanal endorectal dissection can be safely performed using robotic assistance in Centers with advanced minimally invasive expertise in HD management.

## 1 Introduction

The first surgical procedure for the treatment of Hirschsprung's disease (HD) was described by Swenson in 1948 (1). He proposed the resection of the rectosigmoid colon and rectum through a full-thickness dissection, while preserving the sphincter complex (1, 2). Despite its effectiveness, this technique has been associated with injuries to pelvic structures or nerves, resulting in urinary and/or fecal incontinence and sexual dysfunction (2). To reduce these risks, several surgical procedures have been developed modifying the original Swenson operation, all based on the same principle: resecting the aganglionic segment and pulling through the normoganglionic bowel to restore normal bowel function (3).

Since its introduction in pediatric surgery in 2001, robotic technology has shown significant advantages, particularly in pelvic surgery (4). Surgical procedures, such as low rectal dissection, which require enhanced visualization in deep pelvis and precise maneuverability in confined spaces, are especially well-suited to a robotic approach (4, 5).

In this study, we present a monocentric surgical experience with HD patients undergoing rectal surgery in a third-level Pediatric Institute focusing on the evolution of the surgical approach after the introduction of robotic assisted technique. Specifically, we report our initial experience with laparoscopic robot-assisted Restorative Proctocolectomy with minimal transanal endorectal dissection, detailing the technical aspects of this procedure.

## 2 Patients and methods

This study was approved by the Internal Review Board of our Institute (IRB protocol number 0018531/24).

We retrospectively included all patients diagnosed with HD who underwent pull-through surgery at Giannina Gaslini Institute of Genoa between January 2015 and July 2025. A dedicated database was created to collect the following data: gender, age at surgery, length of the aganglionic segment, surgical data, conversion to open surgery, intra-operative events, and short term (<30 day) postoperative surgical complications. For patients who underwent laparoscopic robot-assisted Restorative Proctocolectomy with minimal transanal endorectal dissection, updated follow-up data were also recorded. Postoperative complications were classified according to the standardized Clavien-Dindo classification system.

Robotic technology (Da Vinci XI Surgical System, Intuitive Surgical Inc.; Sunnyvale, California, United States), has been available at our Center for six and a half years (2016, 2020–2025).

**Figure 1:**
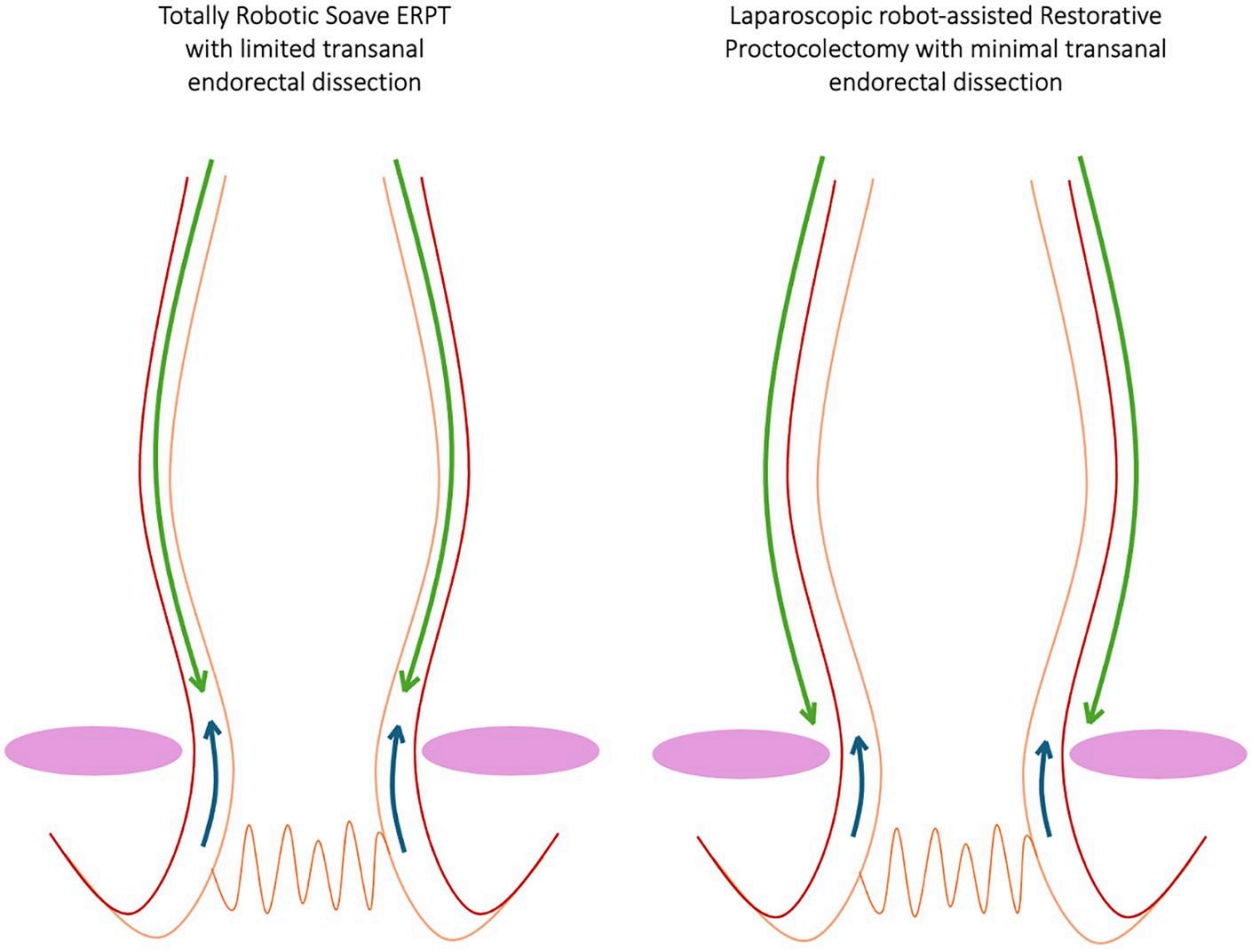
Comparison of intra-abdominal rectal dissection planes between totally robotic soave ERPT with limited transanal endorectal dissection and laparoscopic robot-assisted restorative proctocolectomy with minimal transanal endorectal dissection. The green arrow indicates the intra-abdominal dissection plane, while the blue arrow denotes the transanal dissection plane. The red line represents the seromuscular layer, and the orange line represents the submucosal layer. The pink circles on both sides identify the plane of the levator ani muscles.

All patients underwent one of the following procedures: traditional Soave endorectal pull-through (ERPT); laparoscopic Soave-Georgeson ERPT or Totally Robotic Soave ERPT with limited transanal endorectal dissection (6). Since 2023 we have introduced a new approach: robot-assisted Restorative Proctocolectomy with minimal transanal endorectal dissection. This technique differs in the abdominal phase, where a full-thickness rectal dissection is performed (as in the classic Swenson technique), replacing the submucosal dissection used in laparoscopic Soave-Georgeson ERPT or Totally Robotic Soave ERPT with limited transanal endorectal dissection, while the perineal phase remains unchanged, with a minimal submucosal dissection. The different dissection plane for the two robotic procedures adopted in our Center are highlighted in Figure 1.

Continuous variables were presented with mean, median, minimum, and maximum values. Categorical variables were reported as absolute numbers and percentages. A *p*-value of <0.05 was considered statistically significant.

### 2.1 Laparoscopic robot-assisted restorative proctocolectomy with minimal transanal endorectal dissection

**Figure 2:**
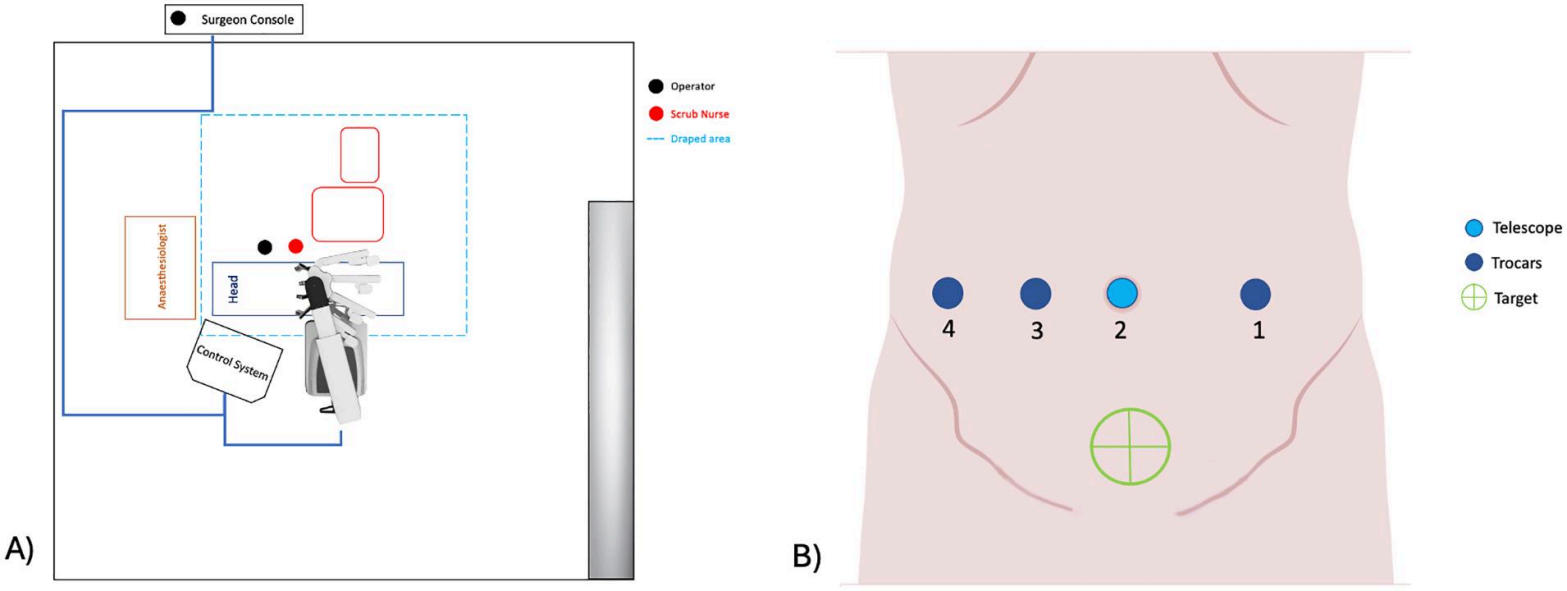
**(A)** operating theatre layout; **(B)** trocar placement.

Figure 2A shows the setting of the operating room. The patient lies in Trendelenburg position, with the left flank elevated. A nasogastric tube is inserted. The patient is prepared and draped circumferentially from the nipples to the feet. A urinary catheter is inserted after the draping is completed.

#### 2.1.1 Abdominal phase

The robotic instruments used include: a) 30° forward-oblique telescope; b) bowel grasper; c) bipolar forceps; d) monopolar device (scissors or hook); e) robotic needle holder. Four trocars are inserted along the transverse umbilical line, with at least 3–4 cm of spacing (Figure 2B). In case of long-segment HD, if a Deloyers procedure is required, the trocars are placed in epigastric region, left and right subcostal areas, and right flank (7).

**Figure 3:**
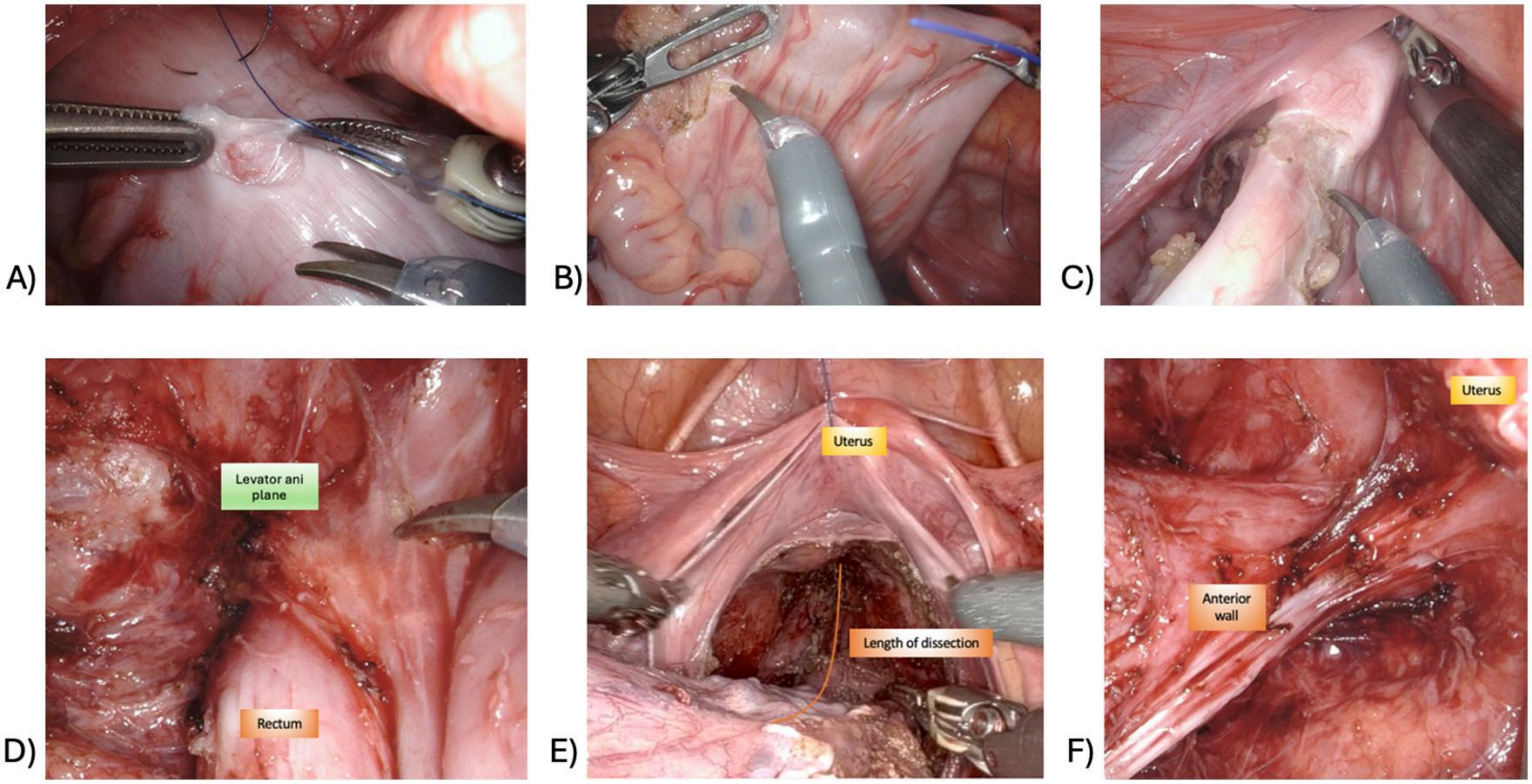
Abdominal phase of robot-assisted restorative proctocolectomy with minimal transanal endorectal dissection. **(A)** Seromuscular biopsy; **(B)** mesorectal and mesosigmoid dissection; **(C)** circumferential incision at the level of peritoneal reflection; **(D)** full-thickness rectal dissection until the elevator ani plane; **(E)** length of the endoabdominal dissection; **(F)** result after endoabdominal dissection (note how anterior dissection is not carried out distally as much as posterior and lateral dissection).

The robot is docked with the pelvis as the target area. A seromuscular biopsy is taken proximal to the transition zone, at the level of dilated colon (Figure 3A). The sample is sent for intraoperative frozen section and histochemical analysis to confirm the presence of ganglion cells. Following adequate mobilization of the mesocolon and mesosigmoid (Figure 3B), rectal dissection is initiated circumferentially at the level of the peritoneal reflection (Figure 3C) and carried caudally with full-thickness dissection (Figure 3D), as described in the classical Swenson technique (2). Dissection proceeds to the level of the levator ani plane (Figure 3E). Care must be taken to stay close to the bowel wall to avoid injury to adjacent pelvic structures. Therefore, in the distal dissection, a robotic bipolar device is preferred, while monopolar instruments may be used proximally. The anterior dissection is deliberately less extensive than the posterior and lateral dissections to reduce the risk of injury to autonomic nerves and avoid damage to the vagina in females or vas deferens in males (Figure 3F). Appropriate tension on the rectosigmoid helps facilitate dissection during this phase.

#### 2.1.2 Perineal phase

After the robotic dedocking, the perineal phase is accomplished with the patient in lithotomy position. A limited transanal submucosal dissection is initiated approximately 5 mm above the dentate line and continued cranially, until the distal rectal segment mobilized during the abdominal phase is reached. The short “muscular cuff” is then incised to enter the peritoneal cavity. Once the ganglionic bowel is pulled through, the anastomosis between the pulled ganglionic bowel and the anus is performed using interrupted 4/0 absorbable sutures. The pull-through is done at least 5 cm proximal to the biopsy site to ensure ganglionated bowel is used for the anastomosis.

## 3 Results

Between January 2015 and July 2025, a total of 65 patients underwent pull-through surgery for HD at our Center. The overall demographic and surgical data of these patients are summarized in Table 1. Fifty-four patients were treated with the traditional techniques previously adopted at our Institution, while 11 patients underwent robot-assisted Restorative Proctocolectomy with minimal transanal endorectal dissection, starting in 2023. Among all patients, 49 had short-segment HD (SSHD), 15 had long-segment HD (LSHD), and one patient was diagnosed with total colonic aganglionosis (TCA). Except for two patients, all others were treated with minimally invasive techniques. Forty-four patients underwent laparoscopic Soave-Georgeson ERPT, eight patients were treated with Totally Robotic Soave ERPT with limited transanal endorectal dissection, these being the surgical procedures of choice in our Center until 2022. Starting from 2023, 11 patients underwent laparoscopic robot-assisted Restorative Proctocolectomy with minimal transanal endorectal dissection.

**Table 1:** Demographic and surgical details of patients with HD who underwent pull-through surgery in our center.

Data	Soave ERPT	Soave-Georgeson ERPT	Totally robotic soave ERPT with limited transanal endorectal dissection	Laparoscopic robot-assisted restorative proctocolectomy with minimal transanal endorectal dissection	TOTAL
*n*° patients	2	44	8	11	65
Age at surgery (months)	17.5 (5–30)	4 (1–242)	31 (3–187)	8 (4–129)	7 (1–242)
Gender	M: 2 (100%) F: 0 (0%)	M: 37 (84%) F: 7 (16%)	M: 7 (88%) F: 1 (12%)	M: 6 (55%) F: 5 (45%)	M: 52 (80%) F: 13 (20%)
Type disease	SSHD: 2 (100%) LSHD: 0 (0%)	SSHD: 36 (82%) LSHD: 8 (18%)	SSHD: 6 (75%) LSHD: 2 (25%)	SSHD: 6 (55%) LSHD: 4 (36%) TCA: 1 (9%)	SSHD: 50 (77%) LSHD: 14 (22%) TCA: 1 (1%)
Redo cases	0 (0%)	3 (7%)	0 (0%)	1 (9%)	4 (6%)
Conversion	0 (0%)	0 (0%) 2 minilaparotomies (4%)	1 (12%)	0 (0%)	1 (2%)
Pre-op ileostomy/colostomy	2 (100%)	4 (9%)	3 (38%)	5 (45%)	14 (21%)
Covering ileostomy	0 (0%)	2 (5%)	1 (13%)	2 (18%)	5 (8%)
Intra-operative complications	0 (0%)	0 (0%)	0 (0%)	0 (0%)	0 (0%)
Short term (<30 days) surgical complications	0 (0%)	4 (9%)	0 (0%)	5 (45%)	9 (14%)

ERPT, endo rectal pull-through; M, male; F, female; SSHD, short segment hirschsprung's disease; LSHD, long segment hirschsprung's disease; TCA, total colonic agangliosis.

Two patients required open Soave ERPT. One had a history of jejunal atresia type IIIB. During the pull-through procedure, a jejunal tapering and revision of the previous jejuno-jejunal anastomosis were necessary. The other was a patient with previously misdiagnosed LSHD, who had undergone multiple open surgeries, including an ileostomy. This case required an open Soave ERPT combined with a Deloyers procedure and ileostomy reversal.

Only one patient required conversion to open surgery due to excessive bowel distension; this occurred in a case of Totally Robotic Soave ERPT with limited transanal dissection. In the laparoscopic Soave-Georgeson ERPT group, two LSHD patients required minilaparotomy to perform a Deloyers procedure because of anastomotic tension. In contrast, among the robot-assisted Restorative Proctocolectomy with minimal transanal endorectal dissection group, two patients also required a Deloyers procedure, which was successfully completed without conversion. At the time of pull-through surgery, 14 patients had a pre-existing stoma. A protective stoma was either maintained or fashioned during surgery in five patients.

A total of four early (<30 days) surgical complications were recorded in the laparoscopic Soave-Georgeson ERPT group. One patient developed toxic megacolon, requiring a diverting ileostomy on postoperative day 3 and subsequently a total colectomy on day 6 due to persistent symptoms.

zne patient developed postoperative anastomotic bleeding requiring surgical revision. Examination under anesthesia confirmed an arterial source, likely originating from the vascular pedicle of the mobilized colon. Hemostasis was achieved with two interrupted 3-0 Vicryl sutures and local application of a hemostatic agent. Another patient developed an anastomotic stricture, identified at the one-month examination under anesthesia, and managed conservatively with serial dilations. The last patient developed anastomotic leak and was re-operated for revision of the coloanal anastomosis, and a diverting terminal ileostomy was fashioned.

Tables 2, 3 report the demographic, surgical and follow-up data of patients who underwent laparoscopic robot-assisted Restorative Proctocolectomy with minimal transanal endorectal dissection. The median follow-up was 10 months (range: 2–33 months). One patient was referred to our Center for a redo pull-through (Patient 8). Intraoperative biopsies revealed a TCA. A laparoscopic robot-assisted Restorative Proctocolectomy with minimal transanal endorectal dissection and ileoanal anastomosis was performed. The child subsequently developed an anastomotic retraction and leak. A surgical revision of the ileoanal anastomosis was performed, and a Penrose drain was placed through the anastomotic site. The patient had a pre-existing covering ileostomy, which had been fashioned prior to and was maintained during the index procedure. Four patients were diagnosed with an anastomotic stricture during the examination under anesthesia performed one month after surgery. All were successfully treated with anal dilatations, and none required a redo pull-through procedure. In addition, one of these patients developed one episode of Hirschsprung-Associated Enterocolitis (HAEC).

**Table 2:** Demographic and surgical details of patients with HD who underwent laparoscopic robot-assisted restorative proctocolectomy with minimal transanal endorectal dissection.

Patients	Gender	Age at surgery (months)	Weight (Kg)	Type of disease	Detailed surgical procedure: Robotic Restorative Proctocolectomy with minimal transanal endorectal dissection	Console time (min)	Operative time (min)
Patient 1	F	15	8.2	SSHD		40	210
Patient 2	M	7	9	SSHD		35	195
Patient 3	M	6	9	SSHD		18	195
Patient 4	F	43	15	LSHD	3× seromuscular biopsies for intraoperative frozen sections (the last one from the pulled-through segment during the perineal phase)	48	310
Patient 5	M	7	7	LSHD	Deloyers procedure + stoma closure	50	160
Patient 6	F	15	9.2	LSHD		57	255
Patient 7	F	46	12	SSHD		50	148
Patient 8	F	129	35	TCA	Redo pull-through. Robotic RP with minimal transanal endorectal dissection with ileo-anal anastomosis	110	385
Patient 9	M	8	7.6	SSHD		40	185
Patient 10	M	4	6.3	SSHD	Stoma closure	35	195
Patient 11	M	4	6	LSHD	Deloyers procedure + stoma closure	45	200

F, female; M, male; SSHD, short segment hirschsprung's disease; LSHD, long segment hirschsprung's disease; TCA, total colonic agangliosis.

**Table 3:** Demographic and follow-up details of patients with HD who underwent laparoscopic robot-assisted restorative proctocolectomy with minimal transanal endorectal dissection.

Patients	Age at follow-up (years)	Type of disease	Short term (<30 days) surgical complications	Post operative complications	Need for post-operative dilatations	Follow-up (months)
Patient 1	3	SSHD			No	33
Patient 2	2	SSHD			No	17
Patient 3	1	SSHD			No	12
Patient 4	4	LSHD			No	12
Patient 5	1	LSHD		AS (CD I), HAEC (CD II)	Yes	14
Patient 6	2	LSHD			No	10
Patient 7	4	SSHD			No	5
Patient 8	11	TCA	Anastomotic leak (CD IIIb)		No	6
Patient 9	0	SSHD		AS (CD I)	Yes	3
Patient 10	0	SSHD		AS (CD I)	Yes	4
Patient 11	0	LSHD		AS (CD I)	Yes	2

SSHD, short segment hirschsprung's disease; LSHD, long segment hirschsprung's disease; TCA, total colonic agangliosis; AS, anastomotic stricture; HAEC, hirschsprung-associated enterocolitis; CD, Clavien-Dindo.

We usually assess postoperative functional outcome according to Wingspread Scoring System (excellent, good, fair, poor). The score could be calculated in only two patients: one had an excellent outcome (Patient 1) and another a good outcome (Patient 4). One patient had a concomitant intellectual disability, and the patient with TCA still has a stoma. All the other patients are not yet toilet trained.

The median age at surgery considering all patients was 7 months. In the laparoscopic group, the median age at surgery was 4 months (range: 1–242 months). In the robotic group, the median age was: 31 months (range: 3–187 months) for Totally Robotic Soave ERPT and 8 months (range: 4–129 months) for laparoscopic robot-assisted Restorative Proctocolectomy with minimal transanal endorectal dissection.

**Figure 4:**
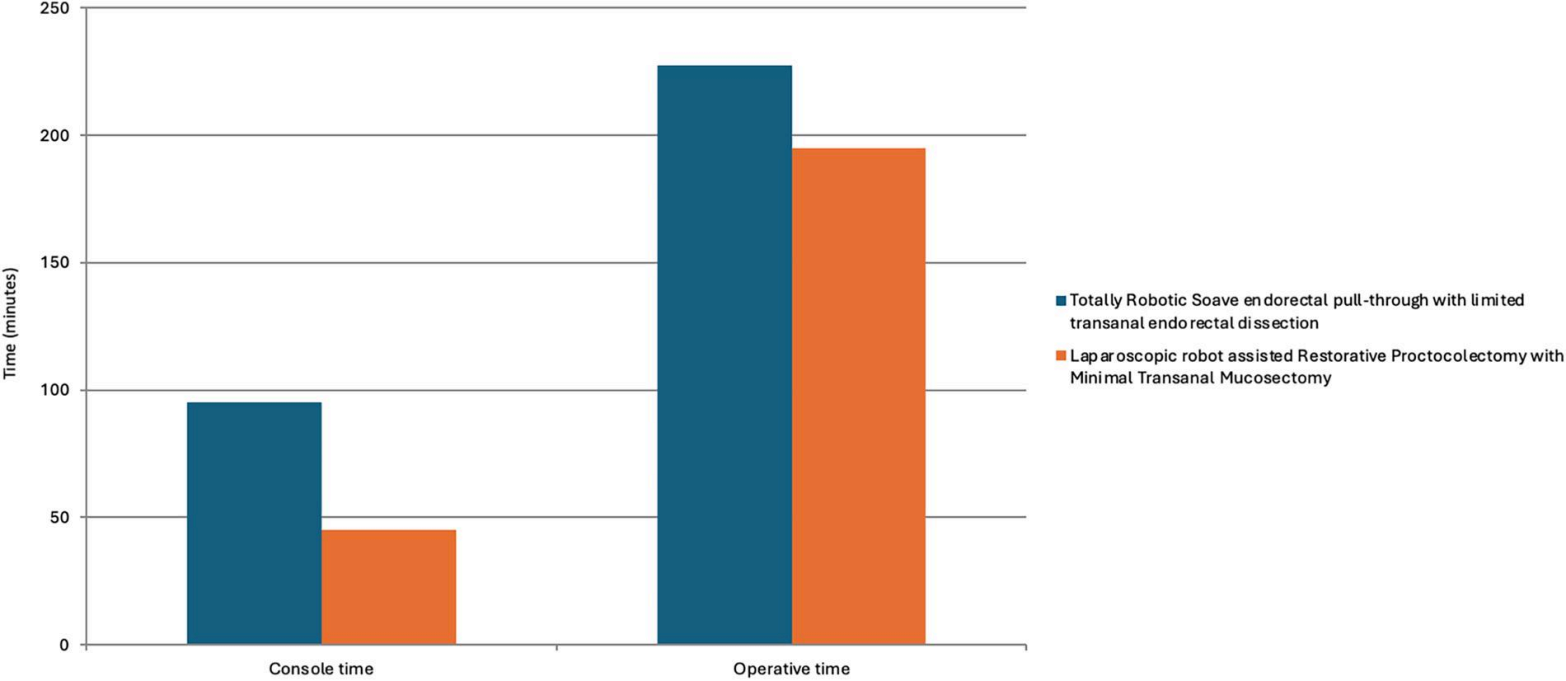
Comparison of the console time and the total operative time between patients who underwent totally robotic soave endorectal pull-through with limited transanal endorectal dissection and patients who underwent laparoscopic robot-assisted restorative proctocolectomy with minimal transanal endorectal dissection.

Comparing the console time between patients who underwent Totally Robotic Soave ERPT and laparoscopic robot-assisted Restorative Proctocolectomy with minimal transanal endorectal dissection (Figure 4), we observed that the console time of the second group was lower (95 vs. 45 min—*p* = 0.003). The difference in total operative time between the two groups did not reach statistical significance (227.5 vs. 195 min—*p* = 0.072).

## 4 Discussion

Several surgical techniques have been described for the treatment of HD. The first one, introduced by Swenson (1), involved a full-thickness rectal dissection with resection of the entire aganglionic segment. The original Swenson surgical technique was delicate and technically demanding, and postoperative complications as urinary and fecal incontinence and impotence were reported (2, 8). To minimize these risks, alternative procedures were developed. Soave (9) proposed a submucosal dissection plane to reduce the likelihood of injury to pelvic nerves and genitourinary structures. With the advent of MIS, Georgeson (10) described the laparoscopic-assisted endorectal pull-through in 1995, which subsequentially became the preferred approach for the treatment of HD in our Center. Following the introduction of robotic technology in our Center, we began performing a Totally Robotic Soave ERPT with limited transanal endorectal dissection, in selected patient with HD (6). This is a modified Soave-Georgeson procedure with an extensive intra-abdominal seromuscular dissection down to 2–3 cm above the dentate line, thereby minimizing perineal operative time since most of the dissection is performed abdominally. The same procedure has been reported by Pini Prato et al. (11, 12), particularly in older children and redo procedures, and by Delgado et al. (13) in patients younger than 12 months. The shorter perineal phase may decrease complications associated with prolonged anal sphincter stretching or deep pelvic dissection, which can negatively affect postoperative continence.

Although these surgical procedures are considered safer in pelvic dissection, they retain an aganglionic muscular cuff, that may cause intraoperative or postoperative complications, such as more intraoperative bleeding (14) or abscess formation (15), or can lead to chronic constipation, recurrent enterocolitis, and failure to thrive (16, 17). Swenson hypothesized that the residual muscular cuff remains in a contracted state, increasing anal canal resistance leading to a functional obstruction (15). Nasr et al. (18) observed reduced enterocolitis rates and a lower incidence of narrowing requiring anal dilatation in patients with shorter cuff. Therefore, the Swenson technique remains the most physiologic, as it involves complete resection of the aganglionic segment without leaving the muscular cuff.

Recent advancements in MIS, particularly robotic surgery, prompted us to reconsider our surgical strategy for HD. In addition to the well-known advantages of laparoscopy, robotic surgery provides image magnification, 3D vision, 7° degrees of motion, tremor reduction and enhanced dexterity. These features make robotic surgery particularly useful for pelvic surgery, especially in children where the narrow field could limit the use of conventional laparoscopic surgery (4, 19). Furthermore, our Center's prior experience with robot-assisted proctocolectomy for ulcerative colitis (UC) and familial adenomatous polyposis (FAP) has contributed to the refinement of our surgical technique and enhanced confidence in performing safe full-thickness rectal dissection. We also observed improved feasibility compared with laparoscopy for the same procedure. Robotic technology offered superior visualization of pelvic structures and precise tissue handling, enabling deep rectal dissection down to the levator ani muscles without pelvic injury or significant bleeding.

Totally Robotic Soave ERPT with limited transanal endorectal dissection was initially adopted in our series for older patients. However, the technique remains challenging due to the submucosal dissection plane and the persistence of the muscular cuff, which may theoretically result in related complications, though none occurred in our series.

Drawing on our experience with robot-assisted restorative proctocolectomy for UC and FAP, procedures that involve full-thickness rectal dissection, we subsequently applied a laparoscopic robot-assisted Restorative Proctocolectomy with minimal transanal endorectal dissection in patients with HD. To date, 11 patients have undergone this procedure without intraoperative complications or conversion to open surgery.

Console time was significantly shorter for the robot-assisted Restorative Proctocolectomy with minimal transanal mucosectomy compared to the Totally Robotic Soave ERPT with limited trasanal endorectal dissection, demonstrating the relative ease and feasibility of full-thickness dissection. This difference likely reflects the fact that full-thickness dissection follows an avascular plane, whereas submucosal dissection requires meticulous identification of the correct layer. Our findings are consistent with those of Deng et al. (14), who reported shorter operative times for laparoscopic restorative proctocolectomy than for the laparoscopic Soave procedure; our results confirm this data using a robotic approach. This finding gains further significance when considering that, among patients undergoing laparoscopic robot-assisted Restorative Proctocolectomy with minimal transanal endorectal dissection, four presented with LSHD and one with TCA. Although the mobilization of the aganglionic colon theoretically requires longer operative time, the console time in this cohort was significantly shorter than in the Totally Robotic Soave ERPT group with limited transanal endorectal dissection, which included only two patients with LSHD. Furthermore, our group successfully performed two robotic Deloyers procedures without conversion, marking a first in the literature (7).

This study primarily serves as a technical proposal. Based on our cumulative experience in surgery in patients with HD and in proctectomy in patients with UC and FAP, we believe that robotic technology enables safer pelvic dissection and minimizes the risk of pelvic injury. Consequently, laparoscopic robot-assisted Restorative Proctocolectomy with minimal transanal endorectal dissection appears to be a feasible and safe option in selected HD patients when performed in specialized Centers with advanced MIS expertise. The youngest patient in our series was 4 months old and weighed 6 kg at surgery.

In literature few authors have already described the use of laparoscopic robot-assisted full-thickness rectal dissection with encouraging results in patients with HD (20–22). A recent multicentric study by Zhang et al. (23) demonstrated comparable mid-term bowel functional outcomes between robotic-assisted proctosigmoidectomy and laparoscopic-assisted Soave pull-through. Hou et al. (22) reported successful robotic Swenson procedures in infants with a median age of 35 days.

Finally, the application of a unified full-thickness rectal dissection technique across different low-volume pathologies may promote surgical standardization and improve expertise, this being a key objective in pediatric surgery, where case numbers are limited.

The main limitations of this study are the small sample size and lack of long-term follow-up for the proposed approach. Further studies are warranted to assess long-term outcomes in HD patients undergoing laparoscopic robot-assisted Restorative Proctocolectomy with minimal transanal endorectal dissection and to better define the subgroups that may derive the greatest benefit from this technique.

## 5 Conclusion

Laparoscopic robot-assisted Restorative Proctocolectomy with minimal transanal mucosectomy could be considered a feasible and safe approach for selected patients with HD, especially when it is performed in high-volume pediatric centers with established expertise in advanced minimally invasive surgery. This surgical approach combines the advantages of an easier abdominal rectal dissection and a shorter perineal phase. Further studies are warranted to assess the long-term outcomes of this technique.

## Data Availability

The original contributions presented in the study are included in the article/Supplementary Material, further inquiries can be directed to the corresponding author.
